# Right Coronary Artery-to-Coronary Sinus Fistula with Giant Tortuous Dilatation of the Right Coronary Artery

**DOI:** 10.5334/jbsr.3546

**Published:** 2024-06-03

**Authors:** Louis Waterloos, Charles Edouard Heylen

**Affiliations:** 1 Université Catholique de Louvain, Bruxelles, Belgium; 2 Radiology department, Cliniques de l’Europe, Avenue de Fré 206, 1180 uccle, Belgium

**Keywords:** Arteriovenous fistula, giant coronary artery, coronary anomaly

## Abstract

*Teaching points:* A coronary artery fistula (CAF) is an uncommon anomaly characterized by a diverse clinical spectrum, ranging from asymptomatic cases to severe complications, including heart failure and myocardial infarction.

## Case History

A 42-year-old patient without a history of cardiac disease or notable medical conditions presented with dyspnea and cardiac palpitations. Echocardiography revealed a pathological dilatation of the origin of the right coronary artery (RCA).

**Figure 1:**
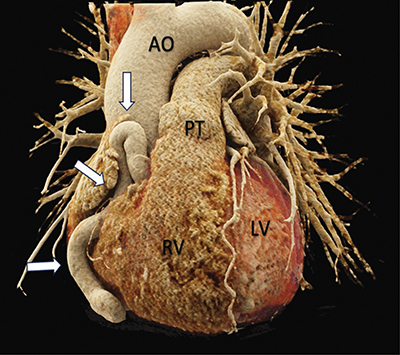
3D rendered cardiac CT-angiography reveals a tortuous dilation of the right coronary artery (white arrows). Front view. *Source:* AO = Aorta, PT = Pulmonary Trunk, RV = Right ventricle, LV = Left Ventricle.

**Figure 2:**
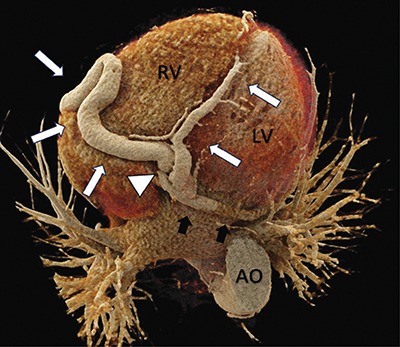
3D rendered cardiac CT-angiography reveals a tortuous dilation of the right coronary artery (white arrows). Near the origin of the posterior interventricular artery, a coronary artery fistula is visible (white arrowhead) that communicates with a moderately dilated coronary sinus (black arrows). Bottom view.

**Figure 3:**
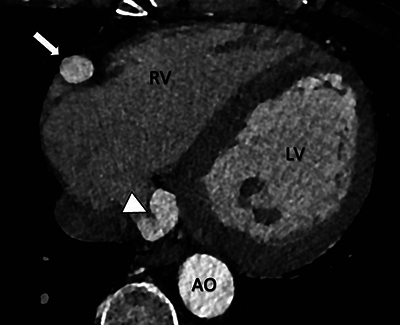
Cardiac CT-angiography reveals a coronary artery-to-coronary sinus fistula (white arrowhead). Note the dilation of the right coronary artery (white arrow). Axial view.

Electrocardiogram (ECG)-gated computed tomography (CT)-angiography revealed a significant tortuous dilatation of the RCA with a diameter of approximately 16 mm (Figures 1–3 white arrows). The dilated and tortuous RCA followed its expected course along the right side of the heart. Near the origin of the posterior interventricular artery, a coronary artery fistula (CAF) was observed (white arrowhead, Figures 2 and 3), communicating with the moderately dilated coronary sinus (Figure 2, black arrows).

## Commentary

CAFs are congenital or acquired abnormal direct vascular communication of coronary arteries with cardiac chambers or any part of the systemic or pulmonary circulation. CAFs are rare and tend to be predominantly asymptomatic [1]. Here, a specific type of CAF is described, known as a coronary artery-to-coronary sinus fistula, which is the third most common variant. The significant and tortuous dilatation of the RCA is attributed to the pressure gradient imbalance caused by the fistula.

The fistula causes a ‘coronary steal phenomenon’ with reduced perfusion of the myocardium. A range of symptoms may manifest, including mild dyspnea, fatigue, angina, congestive heart failure, and even myocardial infarction [1].

Invasive coronary angiography has been the gold standard for assessing CAFs. However, CT angiography has emerged as a valuable modality due to its noninvasiveness, short acquisition time, and superior temporal and spatial resolution. ECG-gated CT angiography, complemented by 3D-reformatting, delivers detailed anatomical insights into CAFs. Radiation exposure is considered as a drawback of CT angiography, as is the limitation to provide hemodynamic information [1].

The therapeutic management of these fistulas depends on their size and the severity of the accompanying symptoms. Small asymptomatic CAFs are typically managed conservatively, with a focus on monitoring for the occurrence of potential complications. In the case of large CAFs, regardless of the symptoms, as well as in cases of small to moderately sized symptomatic, treatment options include surgical ligation or percutaneous transcatheter closure [1].
